# Barriers and facilitators to conducting human subjects research at a safety net institution from the perspective of researchers

**DOI:** 10.1371/journal.pone.0313530

**Published:** 2025-01-08

**Authors:** Sarah J. Barnes, Yewon Na, Mari-Lynn Drainoni, Benjamin A. Linas, Nicholas A. Bosch, Autumn L. Tamlyn

**Affiliations:** 1 Boston Medical Center, Boston, MA, United States of America; 2 Boston University of Chobanian & Avedisian School of Medicine, Boston, MA, United States of America; 3 Boston University School of Public Health, Boston, MA, United States of America; PLOS: Public Library of Science, UNITED KINGDOM OF GREAT BRITAIN AND NORTHERN IRELAND

## Abstract

**Introduction:**

The COVID-19 pandemic revealed glaring problems with clinical research enterprise. Faced with crisis, several trials opened rapidly but enrolled homogenous populations with few Black, Indigenous, and People of Color (BIPOC) individuals. Inclusive trial enrollment is important to inspire trust and confidence in BIPOC populations that have been historically excluded or harmed from research and to improve the generalizability of research findings. Safety-net hospitals and institutions often care for BIPOC populations, and thus it is essential to improve equitable participation in research at these institutions. In this study, we sought to understand barriers and facilitators to research participation at safety net institutions.

**Methods:**

We conducted semi-structured interviews among principal investigators, research assistants, research coordinators, and research nurses who conducted human subjects research at an urban, safety-net hospital from October, 2022 to December, 2022. We used inductive qualitative methods to identify themes associated with barriers and facilitators to clinical research participation.

**Results:**

We completed 28 interviews and identified five themes: (1) compared to non-safety net systems, safety-net systems were perceived to require additional resources and funding to achieve comparable research recruitment and retention; (2) language barriers and translational processes are burdensome for researchers; (3) interactions between research staff and patients impact trust; (4) social determinants of health specific to safety-net populations are a barrier to participation; (5) competing priorities between clinical staff and researchers exist.

**Conclusion:**

Safety net institutions face several barriers to conducting human subjects research. However, identified facilitators may help inform future efforts to reduce inequities in research participation.

## Introduction

The COVID-19 pandemic revealed glaring problems with the clinical research enterprise in the United States (US) [1]. Faced with crisis, several clinical trials opened rapidly but enrolled homogenous populations [2, 3] with few BIPOC (Black, Indigenous, and People of Color) individuals relative to their distribution in the US [4, 5]. Inclusive clinical trial enrollment is important to inspire trust and confidence in BIPOC populations that have been historically excluded or harmed from research and to improve the generalizability of research findings to communities that were disproportionately affected by the pandemic [6–8]. Safety-net hospitals and institutions [9] often care for BIPOC populations, and thus it is essential to improve equitable participant engagement in research at safety-net institutions. We recently examined the perspectives of patients and participants in human subjects research at a safety-net hospital and found that logistical complexities, risk and benefits, recruitment method, credibility of sources, and the informed consent process impacted research participation [10, 11]. However, patient perceptions may not align with the perspectives of healthcare provider and researcher perspectives [12, 13]. In this study, our objective was to identify perceived barriers and facilitators to conducting research at a safety-net hospital from the perspective of researchers.

## Materials and methods

### Study overview

In this qualitative study, we conducted semi-structured interviews of researcher stakeholders and then used inductive analysis to generate themes related to barriers and facilitators to conducting research at a safety net institution.

### Study setting

This study took place at Boston Medical Center (BMC), a private, not-for-profit, safety-net, academic medical center located in Boston, Massachusetts. With a total of, 514-beds, BMC’s mission is to provide consistently accessible health services to all in need regardless of status or ability to pay. The population served by BMC is diverse: 57% of patients are non-white, 30% speak languages other than English, 65% have public insurance [10] and 7% experience unstable housing or homelessness [14].

### Study sample and data collection

Eligible participants were principal investigators (PIs), research assistants, research coordinators, and research nurses at BMC who, in the past 5 years, were involved in studies that required informed consent procedures. We initially recruited participants through purposive sampling PIs across multiple departments, including Infectious Diseases, General Internal Medicine, Endocrinology, Emergency, Obstetrics/Gynecology (OB GYN) and Pediatrics and then identified additional research assistant, coordinators and PI potential participants through snowball sampling (a widely used non-random convenience sampling technique in qualitative research where existing subjects identify additional potential subjects for recruitment based on their social network) [15–17]. When possible, we interviewed both PIs and research staff involved in the same study to understand how perspectives differed based on study team role and level of engagement with study participants. All participants provided verbal consent to participate that was witnessed by a study team member and recorded in a password protected database and received a $50 gift card as compensation for their time.

Each enrolled study subject participated in a 1:1 semi-structured interview via video teleconference. Semi-structured interviews were informed by an interview guide (supplement) which we developed to inquire about the following topics: 1) target study populations; 2) barriers and facilitators to conducting human subjects research at BMC; 3) enrollment challenges 4) experiences with informed consent; and 5) culture of research at a safety-net institution. We recorded participant demographics data retrospectively using an anonymized REDCap-based survey instrument [18]. The study team (S.B., Y.N., N.B., A.T.) met on a bi-weekly basis to review main topics discussed in interviews and discuss the preliminary data. We continued enrollment until thematic saturation was reached.

### Data analysis

Interview recordings were transcribed, cleaned and deidentified prior to analysis. We used an inductive approach to code transcripts. Coding is the process of categorizing transcript excerpts into common patterns. Our coding process started with the internal study team independently reviewing 2 transcripts to guide the creation of preliminary codes (i.e. common patterns and associated definitions). We then iteratively modified and added preliminary codes until the final codebook (supplement) was complete. To reach consensus on codes, each study team member (S.B., Y.N., N.B., A.T.) independently coded 4 transcripts, after which the study team met to discuss and resolve discrepancies. When we reached consensus and finalized codes, each team member single coded a set of transcripts. The internal study team met weekly to discuss differences in coding and resolve discrepancies. We completed all coding in NVivo 12 [19].

Once coding was complete, we used inductive analysis methods [20] to create overarching themes. After identifying preliminary themes, the study team convened to revise preliminary themes into final themes and selected representative quotes for each theme.

The Boston Medical Center and Boston University Medical Campus Institutional Review Board approved all study procedures including the consent process (H-42628).

## Results

### Characteristics of study population

We interviewed a total of 28 participants (11 PIs, 17 research staff) between October 1, 2022 and December 31, 2022. Of our sample, 25 provided demographic information: 16 (64%) of participants identified as female, 8 (32%) of participants identified as male, and 1 (4%) of participants identified as ‘Other’. Approximately half of participants had conducted human subjects research for less than 5 years (Table 1).

**Table 1:** Participant demographics.

Demographics^a^	Cohort (N = 25)
Gender, No. (%)	
Female	16 (64)
Male	8 (32)
Other	1 (4)
Race, No. (%)	
White	18 (72)
Asian or Pacific Islander	3 (12)
Other	1 (4)
Multi-racial or Bi-racial	3 (12)
Hispanic/Latino ethnicity, No. (%)	4 (16)
Age, No. (%)	
21–30	2 (8)
31–40	11 (44)
41–50	3 (12)
51–60	7 (28)
60+	2 (8)

^a^demographics were available in 25 of 28 respondents

### Themes

**Fig 1:**
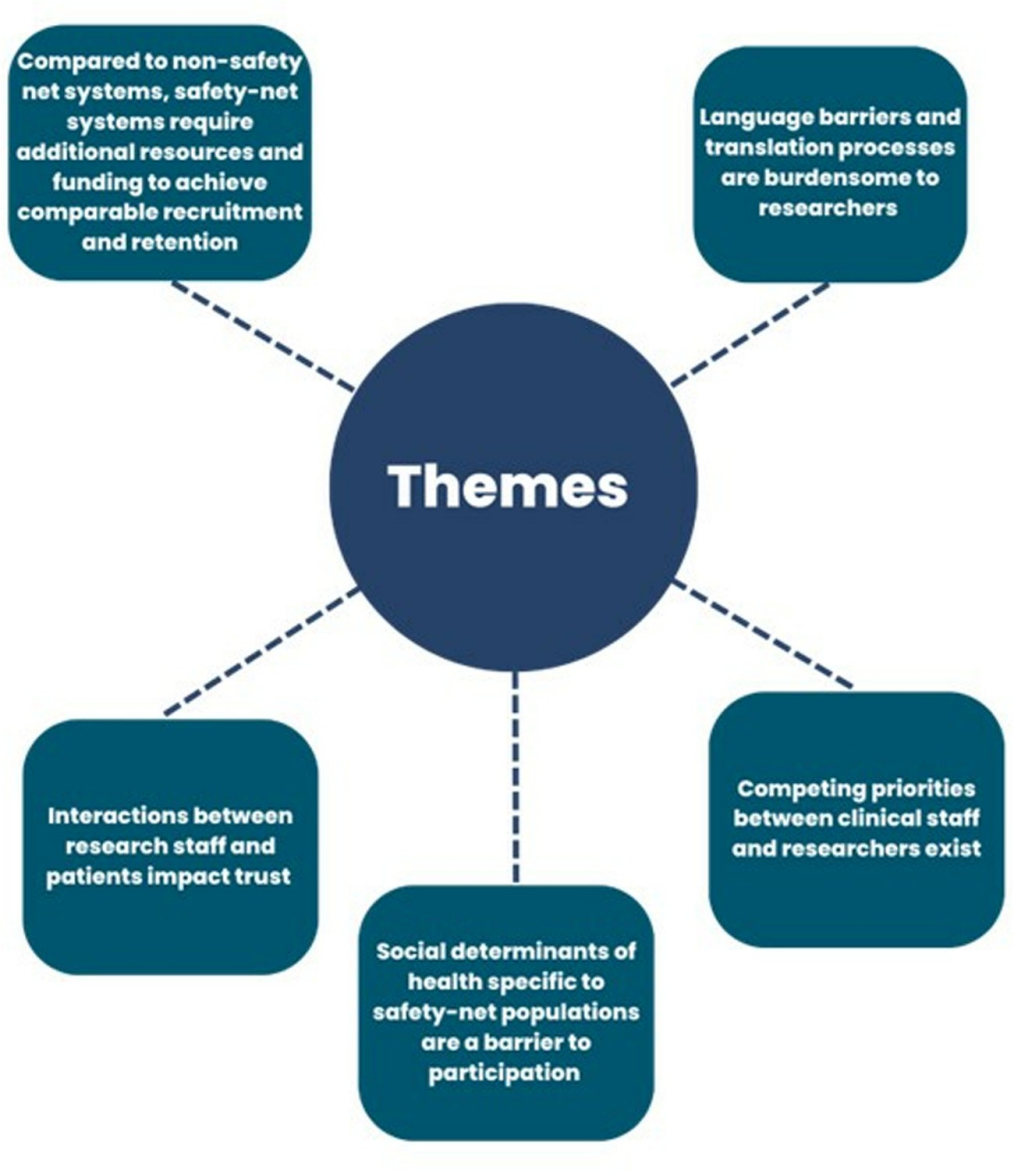
Themes. Shown are themes generated from qualitative analysis of interview transcripts.

We identified five themes from semi-structured interviews (Fig 1 and S1 File).

#### Theme 1: Compared to non-safety net systems, participants perceived that safety-net systems require additional resources and funding to achieve comparable recruitment and retention

Participants perceived that safety-net institutions serve populations who require additional resources, which adds additional barriers to conducting research. One participant stated that the unique circumstances of safety-net populations often make doing research harder: “*Our data does not look like the rest of the world*, *our outcomes are worse up*, *blood pressures are higher*, *our A1Cs are higher*, *our comorbidities and severe adverse events are higher and this has been a three-year long trial*. *It is harder*. *The work that we’re doing in the trial and the effort that we’re putting in to retain these eight patients is much harder*. *There’s a reason that research doesn’t get done in populations like ours*, *because it’s hard”* (Participant 3). Participants indicated that additional resources are often needed to conduct equitable research in a safety-net setting however, research funding and grant systems do not always align: “*So if there were more money to support the research we do to be more inclusive*, *I think that would probably be the most important thing*, *because it’s just like a self-perpetuating issue*, *right*? *Like these populations are historically underrepresented in research and it’s because we just don’t have enough resources to enroll them… the cycle keeps going”* (Participant 8).

Additionally, community engaged research is important to ensuring that research is tailored to the needs of safety-net populations, however, participants expressed that the desire to do longitudinal engaged research with BIPOC communities was limited by current funding strategies: *“And because that’s not how research is funded*, *it’s funded like you have a startup period*, *you have an intervention*, *analysis*, *dissemination*, *[and you] move on*, *and investigators are not incentivized to develop relationships [with partnering communities] because their promotion depends on grants and publications*. *They don’t get rewarded for having strong relationships and being a trusted entity in the community”* (Participant 12).

For multicenter studies, providing equal reimbursement to patients across study sites may actually increase research inequity given the increased resources and funding necessary to enroll and retain participants at safety-net institutions: *"So it’s like equity versus equality*. *Equality is giving everybody the same thing*, *hospital A andhospital B gets $10 per patient they enroll*.*You’re not gonnareach equity with that equal distribution"* (Participant 12).

#### Theme 2: Language barriers and translation processes are burdensome to researchers

Participants reported that a lack of language resources, including translated consent documents, was a barrier to recruiting a diverse population: *“[I] would say that overall it’s challenging to enroll people who don’t use English as their first language… [our] population is much more of a diverse spectrum of languages and that becomes challenging when dealing with industry sponsors and getting them to translate consent and other things for our patients who don’t speak common other languages”* (Participant 1). Participants also viewed the translation process as burdensome: *“By the time that you give the documents you may or may not be able to enroll anyone with that language*. *So let’s say I spoke Vietnamese and they had to translate the document to Vietnamese for our studies*, *we move way too fast so that by the time they finished translating the document*, *we may have a week to continue enrollment and we’re just nowhere near enough to talk to patients about the study and then enroll them”* (Participant 19). Participants identified bi-/multi-lingual study staff as facilitators to language-related barriers: *“Having staff members who would all be bilingual in various languages to be able to try to cover more patients*, *to be able to offer them enrollment*, *I think would increase access into research"* (Participant 20).

#### Theme 3: Interactions between research staff and patients impact trust

Participants highlighted that the interactions between research staff and patients can positively or negatively affect patient trust. One participant reported negative interactions related to the consent process: “*[Consent documents] ‐‐ they’re not super easy to understand*. *I think there’s a lot of scientific information in the consents and then there’s a lot of different things that we have to tell [potential participants] about confidentiality*, *about the IRB*, *about other entities*. *I think that sometimes it can confuse them*. *I have had a couple of occasions where it was just too much*. *It was just too confusing for them and they would say*, *‘Just let me sign it*. *I don’t want to read it and I don’t want you to read it to me because you’re causing me anxiety and I think it’s because of the way it’s written*, *it’s very legal’”* (Participant 15). Strategies to improve trust included using conversational communication techniques: *“letting them [patients] know in advance really is in the interview breaking down the questions*, *being able to rephrase them*, *say them in simpler terms*. *Also letting patients know if you’re not comfortable that’s okay*, *you can skip a question*, *you can go on*. *So they feel more comfortablemaking it conversational… a lot of times it is hard to get someone to open up*. *Especially [for] more sensitive questions about what kind of needs you have*. *Have you had trouble paying your bills*? *Do you have enough food*? *Some people don’t want to admit that to a stranger”* (Participant 13).

#### Theme 4: Social determinants of health specific to safety-net populations are a barrier to participation

Participants noted several social determinants of health that may decrease research participation in safety-net hospitals, including transportation issues, cost associated with participation, and childcare: *“I think one of the barriers community wise for pulmonary hypertension…can be sort of the distance that people [have to go] to come to our center”* (Participant 1), *“I think transportation is huge because if you think about it*, *it costs money to park here*. *And if they take public transportation*, *that’s an expense as well*. *So I think that is significant*, *transportation”* (Participant 15), and “… *some patients just they can’t commit to the research because of other things in their life*, *like they can’t get childcare and they don’t feel comfortable obviously bringing their children into the clinic with them*” (Participant 14). Participants stated that individuals in a safety-net population typically face multiple barriers that may preclude them from participating in research: *“I would say that sometimes recruiting can be really difficult*. *Our patients are very busy*, *have a lot on their plates*, *issues of transportation and childcare*, *and working two jobs and potentially going to school as well*. *And the idea of doing something extra can be really difficult and sometimes just impossible*, *even if people want to participate”* (Participant 20).

In order to help overcome social risk barriers to research, participants suggested that research remain flexible to accommodate the lives of the patients that are being recruited: *“We try to be as flexible as possible in terms of scheduling participant visits before work*, *end of the day*, *weekends*.*We have a study phone where patients can text and call us 24/7*, *so we can try to be flexible when things come up”* (Participant 20). Participants perceived that providing non-monetary reimbursement strategies to participants could potentially facilitate research participation for patients with complex social risks: *"Even things like a voucher for the cafeteria*, *something so they can at least go get a sandwich*, *something that will compensate them for their time*. *They should be rewarded*. *These activities are time consuming*, *so the reward should be in line with the time commitment"* (Participant 24).

#### Theme 5: Competing priorities between clinical staff and researchers exist

In the setting of limited resources, safety-net hospitals may prioritize clinical care over research, leading to potential conflicts involving shared spaces and mission-driven healthcare. Participants expressed challenges in navigating shared clinical and research spaces: “*I think that one of the first things that comes to mind that’s a challenge is space*. *There’s no room to go in and see patients*. *And then there’s such a need for patient care here that there isn’t always time*. *And when I say space*, *I guess I mean time and physical space for people like myself and other members of our team to go in and talk to potential participants just because clinics are busy…*.*"* (Participant 18). Participants further shared experiences of their study teams’ research processes being impeded by clinician and institutional perceptions that research is not part of mission-driven patient care at safety-net hospitals: *“I think BMC has a ways to go in terms of like embracing research*, *at least you know*, *in regards to the rest of their Boston counterparts…I hope that clinicians understand how trusting their patientson enrollment*.*If they’re not actively [with] researchas an option for their patients*, *in informing the care of their patients*,*I think that this will continue to be a barrier"* (Participant 4). Perceived facilitators to overcome competing clinical-research priorities included the need to “*engage the care team members*, *all of them*. *They have to embrace and support the study and advocate for it and make space for it…If there’s not mutual respect and understanding of the entire care team*, *that’s going to be a barrier"* (Participant 12).

## Discussion

We used semi-structured interviews to identify barriers and facilitators to conducting human subjects research at a safety-net hospital from the perspectives of PIs and research staff. Perceived barriers to conducting research included lack of resources and funding, insufficient language resources, mistrust, social determinants of health, and competing priorities between clinical and research staff. Reported facilitators included engaging clinical care team members to create a shared clinical-research vision, hiring research staff with diverse language skills, flexibility with study visit scheduling, and trust building communication techniques. These facilitators could be implemented to improve equitable access to research and combat existing healthcare disparities and may inform future efforts and studies at safety-net hospitals seeking to improve equitable research participation.

Our findings should be considered in the context of prior studies, which have identified research participation perspectives from patients and providers and have examined barriers to research in specific BIPOC populations, but have not identified barriers to research at safety-net hospitals from the perspectives of research teams [10, 11, 21–26]. Common themes identified in our study and the work of others’ focus primarily on interactions between patient participants and study staff, including communication and language barriers [22, 24], social determinants of health specific to safety-net populations [10, 27], and trust in research participation [22, 24, 25]. In both the current study and our prior work [10, 11] interviewing patients and research study participants, issues of trust and mistrust were perceived as major drivers of engagement with research. These concordant findings emphasize the importance of honest, reliable, and positive interactions between participants and researchers to improve research participation Our study also identified several unique barriers to conducting research in the safety-net setting that are relevant to researchers: (1) that the current desire to conduct longitudinal community engaged research at safety-net institutions may not be aligned with short-term ‘study-specific’ funding mechanisms and (2) that equal funding and reimbursement mechanisms for conducting research across study sites in a multicenter study may actually increase research inequities at safety-net institutions where enrollment and retention costs may be greater [28]. Policy makers and researchers should seek to improve equitable (rather than equal) funding and reimbursement of participants for research at safety-net institutions. Participants also felt that patient complexity and comorbidity contributed to difficulties with research processes, especially study retention. In contrast, prior work has primarily focused on comorbidity as a barrier to study enrollment [26, 29]. Future should seek to understand how medical complexity mediates retention and loss to follow-up rates.

To expand upon the existence of social determinants of health among safety-net populations and their impact on ability to participate in research, previous literature has shown that utilizing familiar settings, in-person recruitment, and language concordance can improve participation rates among BIPOC populations [10, 21, 30, 31]. Consistent with our findings, research has also shown that being flexible and adjusting to the needs of underserved populations increases equity in research [10].

Our study has several strengths. We interviewed both PIs and research staff from a diverse set of medical specialties and with a wide variety of past experiences. In addition, we interviewed substantially more individuals than typically needed to reach thematic saturation [32]. This large number and diversity in experiences and expertise increases the likelihood that our study captures perceptions that are broadly applicable to a wide range of stakeholders. Second, by intentionally incorporating queries about facilitators in the interview guide, our study was able to capture not only barriers to research participation at safety-net institutions, but also potential solutions to these barriers that should be implemented and tested in future studies.

This study also has limitations. Our study included participants from a single center. Although the experiences and expertise of participants was diverse, it is not clear that identified barriers and facilitators are unique to BMC alone, safety-net hospitals, or all hospitals who engage in research. Second, recruitment was done through snowball sampling which may miss representation of key informants. Third, our analyses did not distinguish sponsored studies from investigator-initiated studies. Future work is needed to understand how barriers and facilitators of research participation vary based on trial funding and oversight mechanisms. Fourth, our study included researchers at an inner-city safety-net institution. Thus, our results may not generalize to patient populations primarily served by more rural areas, such as certain groups of Indigenous Peoples.

## Conclusion

We used semi-structured interviews to determine barriers and facilitators to human subjects research at a safety-net institution. Barriers to human subjects research focused on issues that may be specific to safety-net institutions (e.g., social determinants of health, lack of language resources, and trust) and issues that may be applicable to all institutions conducting human subjects research (e.g., limited funds and resources, competing priorities between clinical and research staff). While barriers create significant challenges for study teams to overcome, there may be several facilitators to decrease barriers such as: (1) advocating for an increase in resources and funding to promote equitable care; (2) having multi-/bi-lingual staff; (3) building trustworthy relationships; (4) making research flexible to the needs of participants; and (5) ensuring mutual understanding between clinical and research staff. These facilitators should be implemented to improve equitable access to research and combat existing healthcare disparities. The identified barriers and facilitators help to inform efforts at safety-net hospitals focused on reducing inequities in research participation.

## Supporting information

S1 File (PDF)

S2 File (DOCX)

S1 Checklist Interview guide: Barriers to research participation. (DOCX)
