# Correction: Barriers and facilitators to conducting human subjects research at a safety net institution from the perspective of researchers

**DOI:** 10.1371/journal.pone.0338240

**Published:** 2025-12-04

**Authors:** Sarah J. Barnes, Yewon Na, Mari-Lynn Drainoni, Benjamin A. Linas, Nicholas A. Bosch, Autumn L. Tamlyn

The Funding statement for this article is incorrect. The correct Funding statement is as follows: This research was, in part, funded by the National Institutes of Health (NIH) Agreement OT2HL158287. The views and conclusions contained in this document are those of the authors and should not be interpreted as representing the official policies, either expressed or implied, of the NIH.
