# Supplementary figures and images for: Barriers and facilitators to conducting human subjects research at a safety net institution from the perspective of researchers
Source: PLoS One. 2025 Jan 8;20(1):e0313530. doi: 10.1371/journal.pone.0313530 (PMC11709291; doi:10.1371/journal.pone.0313530)

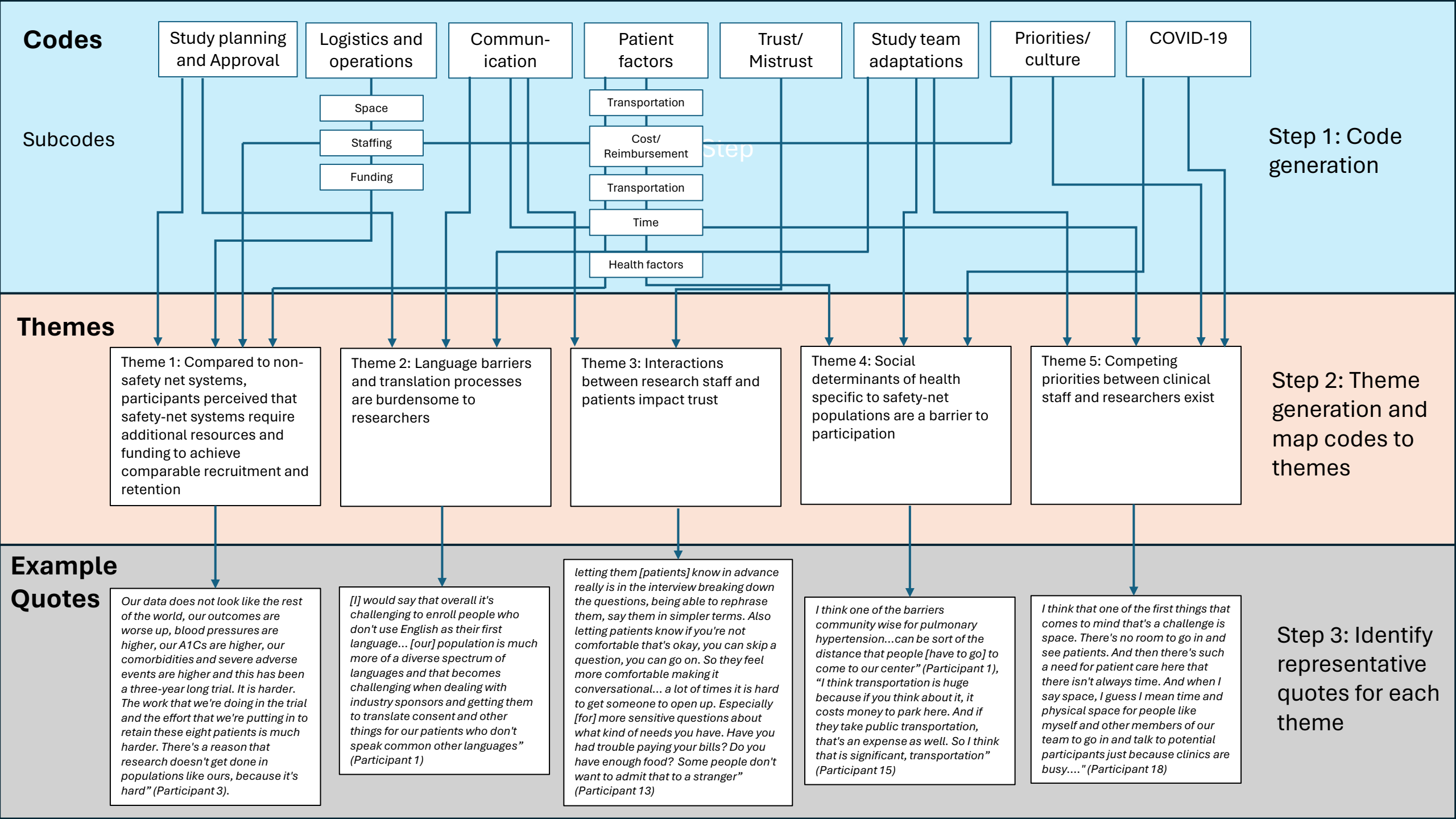

Supplement: S1 File — (PDF) [file pone.0313530.s001.pdf]
