# Supplementary material for: Barriers and facilitators to conducting human subjects research at a safety net institution from the perspective of researchers
Source: PLoS One. 2025 Jan 8;20(1):e0313530. doi: 10.1371/journal.pone.0313530 (PMC11709291; doi:10.1371/journal.pone.0313530)
Supplement: S2 File — (DOCX) [file pone.0313530.s002.docx]

| Code | Definition | Barriers (inclusion/exclusion) | Facilitators (inclusion/exclusion) |
| --- | --- | --- | --- |
| 1. **Study planning and approval** | Discussion of logistical and operational aspects of a research study **that occur prior to starting enrollment** including study planning, study setup, IRB processes, and other pre-study activities. | Include: Statements such as, *“One of things that's very frustrating to me is the lack of transparency in IRB criteria.”*  Exclude: Statements relating to adaptation by study team to improve access to research, code to Study team adaptations. Exclude statements related to discussing consent with a participant, code to Communication. | Include: Include statements referring to logistical or operational systems that have supported pre-enrollment research activities, such as, *“I think the individual IRB analysts are facilitators and prior to submitting the application, I always start talking with analyst, which helps me get a couple steps ahead.”*  Exclude: Exclude statements alluding to research staff that have utilized different strategies to support research, code to Study team adaptations. |
| 1. **Logistics and operations** | Discussion of logistical and operational aspects of conducting a research study **that occur after starting enrollment**, including routine operations, study staff processes, and institutional processes. | Include: Include statements such as, *“in terms of timing a visit it can be difficult because a lot of these clinical research groups will work on a variety of different trials. So they have a bunch of people that they're looking to appease and to incorporate into their schedule and sometimes maybe they're also overworked as well.”*  Exclude: Exclude statements referring to logistical barriers that occurred before study approval, such as, *“We wanted to be able to send text messages with patients and it took two years to get this approved due to logistical barriers”* Code to Study planning and approval. | Include: Include statements pertaining to institutional resources that have helped to conduct research, such as, *"The grants office has been fantastic, if I send an email to the person that I've worked with the most, it's like the floodgates open of resources and ideas and they provide a timeline.”*  Exclude: Exclude statements referring to logistical facilitators that took place before study approval, such as references to IRB approval, code to Study planning and approval. |
| - 1. **Space** | Logistical or operational references in regards to space required to conduct a research study. | Include: Include statements such as, *“And generally, we need space to be able to conduct our studies. And it really affects the type of studies I'm able to take on.”*  Exclude: Exclude statements that refer to challenges of limited time to conduct research in shared spaces, code to Funding. | Include: Include statements about how having secure research space can help to conduct research, such as, *“I think it's helpful when the research staff is embedded in the clinical setting where you receive care. If you're like right there, it's not like somewhere separate you have to travel. We're right there, you just have to walk down the hall, and there we are.”*  Exclude: Exclude statements that describe strategies or systems that the study team has built to secure space for research activities, code to Study team adaptations. |
| - 1. **Staffing** | Logistical or operational references in regards to staffing required to conduct a research study. | Include: Include statements pertaining to barriers to hiring, maintaining, and other research staffing barriers, such as, *“when I go to hire an RA or a data analyst or whomever, it all goes through HR ... and the salaries are very low”*  Exclude: Statements such as, *“We haven't figured this out, but the other barrier was having research staff that spoke languages other than English.*” Code to Communication. (Rationale: although concerning study staff, the major barrier relates to language. However, if the example included that multilingual study staff were too expensive to hire, this would fall under staffing category). | Include: Include statements referring to staff skills that have supported research activities, such as, “It’s nice to have someone that knows the ins and outs of the hospital system...”  Exclude: Exclude statements referring to helpful strategies that research staff have used to conduct/support research, code to Study team adaptations. |
| - 1. **Funding** | Logistical or operational references in regard to funding mechanisms and funds to be able to conduct a research study. | Include: Include statements such as, *“For me pregnancy research happening at the [BMC affiliated institution] where people have protected time to write grants and do the bio stats and things through methodology etc. Whereas we're all doing clinical sessions a week trying to grind it out.”*  Exclude: Exclude statements of funding issues when hiring/for adequate staffing, code to Staffing | Include: Include statements alluding to how adequate funding and allocated time for research have supported research activities, such as, *“in GIM, one of my collaborators is a physician in GIM who has a lot of protected research time and we launch projects together, and it makes it possible for me to take a small amount of research time and do a lot.”*  Exclude: Statements such as *“The support we receive from the Grants Office enhances our ability to conduct research,”* code to Logistics and Operations. |
| 1. **Communication** | References related to communication with participants including; dialect, speech, language spoken, language of materials, visit reminders, consent processes, etc. | Include: Include statements referring to things that acted as a barrier to communication i.e., *“Guess another barrier is, in our consent, because the app is only in English and the surveys, each visit survey they have is only in English. We can only target English speakers”*.  Exclude: Exclude statements referring to challenges with IRB set up or creation of consent forms, code to Study set-up. | Include: Include statements pertaining to things the improved communications, such as, having a study team member that spoke another language and/or interpreter services. Include references to the consent processes that went well.  Exclude**:** Exclude statements regarding the location where consent takes place, code to Space. |
| 1. **Patient Factors** | Discussion of factors related to conducting research from the perceived patient perspective. | Include: Statements related to a patient factors that impacted their ability to participate in research (that does not fall into one of the child codes), such as *“it can be challenging to recruit the BMC patient population due to socioeconomic factors.”*  Exclude: Statements related to providing transportation to patients, code to Transportation. | Include: Statements related to patient health factors that improved their ability to participate in a research study (that does not fall into one of the child codes below), such as *“The BMC patient population is always willing to join when I recommend a study.”*  Exclude: Statements such as*, “I find participants do not want to join a study if the compensation is not sufficient*” Code to Cost/reimbursement. |
| - 1. **Transportation** | References to transportation to and from study visits or other transportation needs of participants. | Include: Comments about what made transportation hard for individuals, such as “*the study location was far away and people couldn’t get there”.*  Exclude: Exclude statements pertaining to operational barriers to research such as trying to schedule time with staff, code to Logistics and operations. | Include: Include statements of how receiving or having transportation support helps individuals in research participation, such as, *“I think another big one that we recently started to do is support for travel, so like rural health or whatever compensation, or being able to Uber somebody to and from their recent appointment has been invaluable for the studies we've been able to do that.”*  Exclude: Exclude comments referring to study team initiatives when it comes to getting participants to study locations, code to Study team adaptations. |
| - 1. **Cost/reimbursement** | References to cost of or reimbursement for participating in research. | Include: Include statements alluding to how compensation or reimbursement may act as a barrier to research participation (i.e., not enough compensation).  Exclude: Exclude statements referring to participant time constraints that limit their ability to participate, code to Time. | Include: Include statements alluding to how compensation or reimbursement helps individuals to participate, such as, *“... finance is one thing that I think gives people a positive response.”*  Exclude: Exclude statements that describe processes to secure adequate compensation for participants, code to Study planning/approval. |
| - 1. **Time** | References to the duration of time required to participate in research studies and its impact on willingness to participate. | Include: Statements referring to difficulty conducting/completing studies due to time constraints which refer to patients being unable to make appointments, not being able to set aside time to participate in the study, etc.  Exclude: Statements that refer to study approval timeline (i.e. statements referring to the IRB taking too long). Code to Study planning/ approval. | Include: Statement such as, *“it does not take very long to participate and therefore we find patients are willing to take a few extra minutes to be a part of the study.”*  Exclude: Statements related the time it takes to recruit participants for a study, code to Logistics and Operations. |
| - 1. **Patient Health Factors** | References to patient/participant health conditions that impact their ability to participate in research studies. | Include: Include statements about how participants having a substance disorder can be challenging, such as, *“I really have to be careful about some of the population in terms of the setting I enrolled because…a lot of people are under the influence of substances…”*  Exclude: Statements referring to strategies used by the study team to enroll participants despite barriers. Code to Study team adaptability/innovations. | Include: Statements regarding a health factor that increases a participant’s wiliness to be in a study, such as *“This particular population is eager to seek treatment for their health condition and therefore eager to participate in a study that may support their recovery.”*  Exclude: Exclude statements referring to what the study team does to navigate consenting or conducting research activities with participants who have underlying health conditions or use substances, code to Study team adaptations. |
| 1. **Trust/Mistrust** | Discussion of perceived patient feelings of trust/mistrust as related to participating in a research study that reflects their willingness to participate or not to participate. | Include: Include statements referring to participant hesitancy to participate in research despite measures to protect participants, such as *“There's confidentiality and privacy concerns even though we keep all the information anonymous and everything…”*  Exclude: Exclude statements referring to participants not having free time to participate in research, code to Time. | Include: Include statements referring to research participants showing trust and willingness to participate, or positive perceptions to participating in research.  Exclude: Exclude research study team strategies that have allowed them to successfully recruit in the community, code to Study team adaptations. |
| 1. **Study team adaptations** | Changes and strategies that have been implemented by the study team. These can be unwanted or wanted adaptations. | Include: Include statements related to study team adaptations that were unwanted but necessary, such as those imposed by regulatory groups that resulted in changes to the study design or processes.  Exclude: Exclude statements referring to staffing barriers that study teams experience when conducting research, code to Staffing | Include: Include statements referring to strategies and systems the study team used to support recruitment, retention, and other research study processes, such as, “*... the newly diagnosed patients, they're processing everything and so it's great just to listen to them and show empathy into what they're going through and that they are in a safe space…”*  Exclude: Exclude statements about changes in staffing in the study team that have supported/improved study team strategies, code to Staffing. |
| 1. **Institutional priorities/ Culture** | The research staff’s perspective of the institution’s prioritization of research and things that are being done (or not done) to facilitate research culture. | Include: Include statements that mention the lack of research culture at BMC, such as *“Where your point of contact is the CMO, the CMO of some community health center is not going to be, you're not gonna be on the top of their priority list.”*  Exclude: Exclude statements referring to spacing issues, code to Space. | Include: Include statements referring to research culture at BMC such as, *“...many good initiatives are happening at the institutional level with opportunities to touch on that those doors are opening in a really positive way. I have come to the Equity Partnership Network, I think that the accelerator is putting it together.”*  *Exclude:* Exclude statements about distrust of the research community, code to Trust/Mistrust. |
| 1. **COVID-19 effects** | Research impacts (positive or negative) that were perceived as being due to the coronavirus 2019 pandemic. | Include: Phrases related to COVID-19’s impact on recruitment, retention, research infrastructure, etc. in relation to barriers to research. Examples might include changes from face-to-face to remote consenting because of the risk of study team infection that resulted in lower enrollment rates.  Exclude: statements referring to trust/mistrust that may have become more prominent during the COVID-19 pandemic (e.g., distrust of vaccines) but were not a direct result of the pandemic. Code to Trust/Mistrust. | Include: Include phrases related to COVID-19’s impact on recruitment, retention, research infrastructure, etc. in relation to facilitators to research, such as, increases in the institutional capacity to do research related to recognition during the COVID-19 pandemic that BMC was ill-equipped to engage its patients in research.  Exclude: Exclude statements referring to changes made by the research team in response to the impacts of COVID-19, code to Study team adaptations. |

**Barriers and Facilitators may include both known facilitators and perceived facilitators
