# Supplementary material for: Barriers and facilitators to conducting human subjects research at a safety net institution from the perspective of researchers
Source: PLoS One. 2025 Jan 8;20(1):e0313530. doi: 10.1371/journal.pone.0313530 (PMC11709291; doi:10.1371/journal.pone.0313530)
Supplement: S1 Checklist — (DOCX) [file pone.0313530.s003.docx]

Interview Guide: Barriers to research participation

Cohort: PI’s and study staff from Boston Medical Center

Introduction: Today we will be discussing conducting research with patients of Boston Medical Center, focusing on aspects that either help or hinder research when engaging the Boston Medical Center patient population. As a safety-net hospital with a diverse patient population, we are interested in learning more about your thoughts and experiences conducting research in this context. For the purposes of this study, we are defining research studies as those which meet IRB definitions as human subjects research, required a full IRB review, and required participants to undergo full informed consent procedures. We are interested in learning about your experience as it relates approximately to the past five years, to capture pre and post-COVID experiences.

1. How long have you worked for BMC? How many of these years have you been working in research?
   1. What role/roles have you played in the research you have done (i.e. PI, research assistant, administrative support)?
2. Can you tell us about the human subjects research studies you’ve conducted with BMC patients in the last ~5 years?
   1. Probe about what kinds of studies are done, where study activities take place (e.g. clinic, GCRU/independent, remote)?
3. For the above studies, please tell me about the patient populations you aimed to enroll (e.g., demographics)?
   1. Why was this the patient population you prioritized for your research?
   2. Have there been any patient populations you ideally wanted to enroll but couldn’t? If so, why?
4. What barriers to conducting research at BMC have you experienced?
   1. What were the consequences of these barriers to your research (study design, populations studied, study reach)? To what extent did this impact your work?
   2. Probe about structural/institutional barriers (e.g., IRB policies, BMC research policy, research operations, consenting requirements, language support, research professionals network, GCRU, CTO, CTSI, CRN, LABS core, IT, Research Information Services, general counsel, BMC Development, BMC interpreter service)
   3. Probe about recruitment from Community Health Centers (CHC’s) If you have recruited from CHC’s, were there any regulatory barriers that impacted your work?
   4. Probe about patient-specific barriers (e.g., language barriers, trust in healthcare/medical research, understanding of research, etc.)
   5. Probe about barriers to conducting community-engaged research (e.g., staffing barriers, barriers to community access, barriers to meet study timelines)
   6. Probe about recruitment and retention (both patient specific and institution wide)
   7. Were you ever unable to successfully launch or complete a study because of barriers to conducting research at BMC and/or with BMC patients? If so, please tell me about that?
5. Can you provide an example anecdote of a time when enrolling a patient at BMC was challenging?
   1. What, if anything, did you do to overcome the challenges?
   2. Based off this particular experience, is there anything you did (or would do) differently when enrolling subsequent patients?
6. In your experience, what are the facilitators to or strategies for conducting successful research at BMC?
   1. In what ways have these facilitators/strategies helped you conduct research with BMC patients?
   2. Probe about individual strategies (i.e. recruitment tactics, retention strategies, study team practices) as well as institution wide facilitators (e.g., IRB policies, BMC research policy, research operations, consenting requirements, language support, research professionals network, GCRU, CTO, CTSI, CRN, LABS core, IT, Research Information Services, general counsel, BMC Development, BMC interpreter service)
   3. What facilitators (existing or desired) do you believe are the most important for engaging BMC patients in research?
7. For the studies you have conducted, can you discuss your experiences with the informed consent process?
   1. How successful do you feel you and/or your team are at obtaining informed consent from BMC patients?
   2. Probe about perception of participant understanding
   3. Probe about barriers to obtaining informed consent (language barriers, exchange of forms/signatures, use of technology, etc.)
   4. Probe about facilitators of informed consent (i.e. study staff who are representative of target population demographics culturally/racially/linguistically, using interpretive services)
8. Do you have any specific reflections about conducting research at a safety-net hospital that we have not already discussed?
   1. If you could change something, what would you change?
   2. Probe about additional barriers/facilitators/strategies
   3. Probe about culture of research in the context of a safety-net hospital
